# The effect of spermine on *Tetranychus urticae-Cucumis sativus* interaction

**DOI:** 10.1186/s12870-023-04573-5

**Published:** 2023-11-17

**Authors:** Shima Shahtousi, Ladan Talaee

**Affiliations:** Department of Plant Protection, College of Agriculture, Isfahan University of Technology, Isfahan, 84156-83111 Iran

**Keywords:** Life table, Polyamine, Plant defense, Oxidative stress, Plant–herbivore interaction

## Abstract

**Background:**

Two spotted spider mite, *Tetranychus urticae* (Acari: Tetranychidae) is one of the most important plant pests in the world. Due to increased resistance of mites to acaricides, it is necessary to use other methods such as inducing resistance in plants by natural compounds for pests' management. Polyamins such as spermine are effective in increasing plant resistance to biotic and abiotic stressors. In this research, the effect of spermine treatments in cucumber plants on life table parameters of *T. urticae* was investigated. Also, top-down effect of spermine and *T. urticae* on cucumber biochemical parameters was measured. In the experiments, 1, 2 and 3 mM spermine concentrations were used.

**Results:**

Amongst the spermine treatments, those mites that fed on cucumbers which received 1 mM spermine showed the shortest protonymphal period and higher ovipositon period, fecundity, gross and net reproductive rates and life expectancy compare to control. Treatment with 2 mM spermine lead to the longest teleochrysalis period and shortest range of age-stage-specific fecundity period. In addition, 2 mM spermine lowered intrinsic and finite rate of population increase in *T. urticae*. The longest larval period of *T. urticae* was observed in 3 mM spermine. Feeding of *T. urticae* from cucumber plants increased hydrogen peroxide (H_2_O_2_), malondialdehyde (MDA) content, electrolyte leakage (EL) level and ascorbate peroxidase (APX) activity but inhibited catalase (CAT) activity in this plant. Infested cucumber plants treated with 2 mM spermine showed lower H_2_O_2_ and MDA content and highest activity of APX and CAT on day 1 and 3 compare to the others. The 3 mM spermine increased H_2_O_2_ content in infested plants during the whole experiment as well as non-infested plants in day 5 and 9 only. This treatment induced the highest MDA content and lowest catalase activity on day1, 3 and 5 of experiment in infested plants.

**Conclusion:**

This study showed that 2 mM spermine was the only effective concentration that reduce cucumber sensitivity to *T. urticae.* The trend of changes in biochemical parameters, especially H_2_O_2_, in 3 mM spermine was abnormal, and this concentration could be considered toxic.

**Supplementary Information:**

The online version contains supplementary material available at 10.1186/s12870-023-04573-5.

## Background

Tetranychidae family comprises more than 1,300 species of which about a hundred are considered plant pests and about ten of them are considered major pests [42]. Two-spotted spider mite, *Tetranychus urticae* Koch, is one of the major pests which is globally distributed and has host range of over 1100 plant species [1]. This mite is a phytophagous pest causing significant damage to various fields, gardens, greenhouses and ornamental plants [57]. Both adult and immature mites feed from the contents of mesophyll cells and create pale and white spots on the leaf surface. *T. urticae* covers the plant with webs and causes leaves to fall, reduces flowering and decreases the quality and quantity of products [7].

Over time, plants and herbivores acquire a variety of mechanisms to face each other. Defense mechanisms can be induced in plants by pests like *T. urticae* [63]. This mite can induce or suppress plants' defense by creating wounds, producing saliva, oviposition fluids, feces, webs and exuviae^.^ Detection of mite attack in plants is done via damage-associated molecular patterns which are plant derivatives, or through herbivore-associated molecular patterns which are related to mites [8]. After binding these peptides to pattern recognition receptors, depolarization of cell membrane, cytosolic Ca^2+^ influx and production of reactive oxygen species (ROS) occurs [34].

ROS include compounds such as singlet oxygen (O_2_), superoxide radical (O_2_^−^), hydroxyl radical (OH^−^) and hydrogen peroxide (H_2_O_2_) [47]. The ROS level especially H_2_O_2_ increases when plant is under stress. H_2_O_2_ acts as a dual sward; it damages biological molecules such as nucleic acids, amino acids, proteins and lipids causing lipid peroxidation and cell death. On the contrary, H_2_O_2_ acts as a signaling molecule creating an appropriate response to the plant stress [52]. Therefore, it is very important to maintain the balance between the production and elimination of ROS in plants. To achieve this, plants have enzymatic and non-enzymatic antioxidant systems. Enzymatic antioxidants include superoxide dismutase (SOD), ascorbate peroxidase (APX), guaiacol peroxidase (GPX), catalase (CAT), dehydroascorbate reductase (DHAR) monodehydroascorbate reductase (MDHAR), glutathione reductase (GR), and glutathione-S-transferase (GST) [44]. Glutathione, ascorbate, proline, α-tocopherol, carotenoids and flavonoids are non-enzymatic antioxidants, also involved in ROS scavenging [16].

Polyamines are low weight polycationic molecule compounds with two or more amino groups. The most important plant polyamines are putrescine, spermidine and spermine. These compounds form a new group of plant growth regulators which participate in plant development and physiological processes such as cell division, embryogenesis, flowering, senescence and stress response [14]. Spermine has a higher number of amino groups and creates a higher physiological activity in plants compared to putrescine and spermidine, and for this reason, it has received more attention [14]. This polyamine works as an intermediary compound for modulating plant defense response to biotic and abiotic stresses. Spermine regulates different oxidative and hormonal signaling pathways that are suitable for optimal defense response to various stressors [59]. This compound accumulates in response to biotic and abiotic stresses, but deals with them in two different ways. When exposed to abiotic stress, spermine inhibits the production of ROS by hormonal regulation and inducing enzymatic and non-enzymatic antioxidant responses. In response to biological stress, this polyamine can induce more ROS production via mitochondrial membrane dysfunction (expression of genes related to hypersensitivity response) and hormonal regulation [53, 60].

In recent years, empowering plant defense system to overcome different biotic and abiotic stresses, including herbivorous insects, has become a center of attention. Induced resistance by natural compounds is an integrated or alternative method to other approaches for pest control such as chemical pesticides [9, 37]. Many natural and synthetic compounds are known to activate the defense response against a specific type of biotic or abiotic stress, but there are very limited compounds that work on a wide range of stresses [2]. Considering the growing interest in use of polyamines against abiotic and some biotic stresses such as fungi, nematodes and viruses, it seems necessary to investigate the effect of these compounds on herbivorous arthropods. This study aimed to explore the effects of different concentrations of exogenous spermine on cucumber antioxidant responses against two spotted spider mite over time and the performance of this pest.

## Results

### The effect of spermine and *T. urticae* on cucumber H_2_O_2_ content

**Fig. 1:**
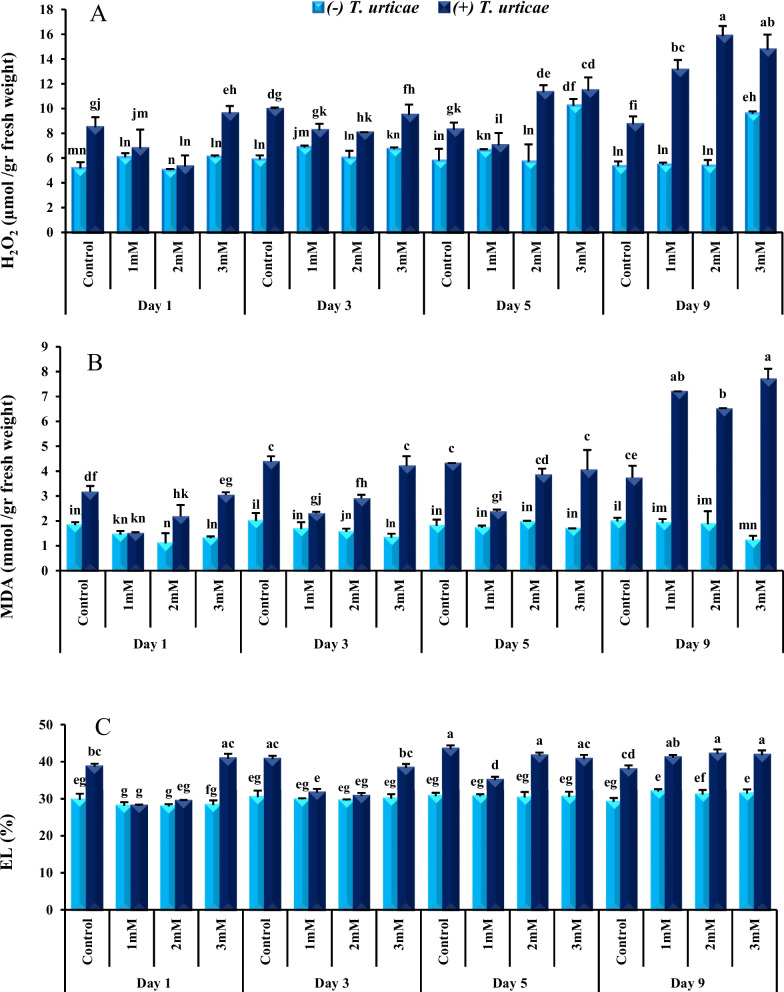
The contents of hydrogen peroxide (H_2_O_2_) (**A**), malondialdehyde (MDA) (**B**) and electrolyte leakage (EL) (**C**), in Spadana cucumber leaves in response to *Tetranychus urticae* and different spermine concentrations (1, 2 and 3 mM) treatments. Mean (± SD) was calculated from three replicates for each treatment. Bars with different letters are significantly different at *P* ≤ 0.05 applying LSD test

The Fig. 1A shows that mite infestation increases H_2_O_2_ concentration in cucumber leaves. Plants infested with mites and treated with 2 mM spermine showed lower H_2_O_2_ level at day one whereas infested plants treated with 3 mM spermine failed to demonstrate the same effect. The results in day 3 were similar to day one. Plants treated with 2 mM and 3 mM concentrations of spermine and in presence of mite on day 5 and 9 of exposure showed significant increase in H_2_O_2_ levels compared to the control plants exposed to mite. The highest level of H_2_O_2_ observed in infested plants treated with 2 mM and 3 mM spermine on day 9. There is no significant difference in H_2_O_2_ level between plants without mite in different groups on day 1 and 3 but H_2_O_2_ was higher in plants without mite treated with 3 mM spermine on day 5 and 9. H_2_O_2_ level in plants without mite treated with 3 mM spermine is similar to plants with mite treated with 2 mM and 3 mM spermine on day 5. Cucumber plants bearing mites treated with 1 mM spermine for the first time showed high level of H_2_O_2_ on day 9.

### The effect of spermine and *T. urticae* on cucumber MDA content and EL level

The presence of mites increased both MDA (Fig. 1B) and EL (Fig. 1C) levels in cucumber plants. Spermine at 1 and 2 mM concentrations suppressed MDA and EL levels in + *T. urticae* plants to the same level as -*T. urticae* plants on day 1 and 3. Spermine at 3 mM concentration in presence of mites showed higher levels of MDA and EL and similar to control plants + *T. urticae* on day 1, 3 and 5. On day 5, only 1 mM spermine could suppress MDA and EL in + *T. urticae* plants. On day 9, MDA and EL contents increased in all spermine concentration treatments + *T. urticae* compared to control plants + *T. urticae* and reached their highest value during the experiment. On this day, the EL values of the three treatments were even higher than control + *T. urticae*.

### The effect of spermine and *T. urticae* on cucumber APX activity

**Fig. 2:**
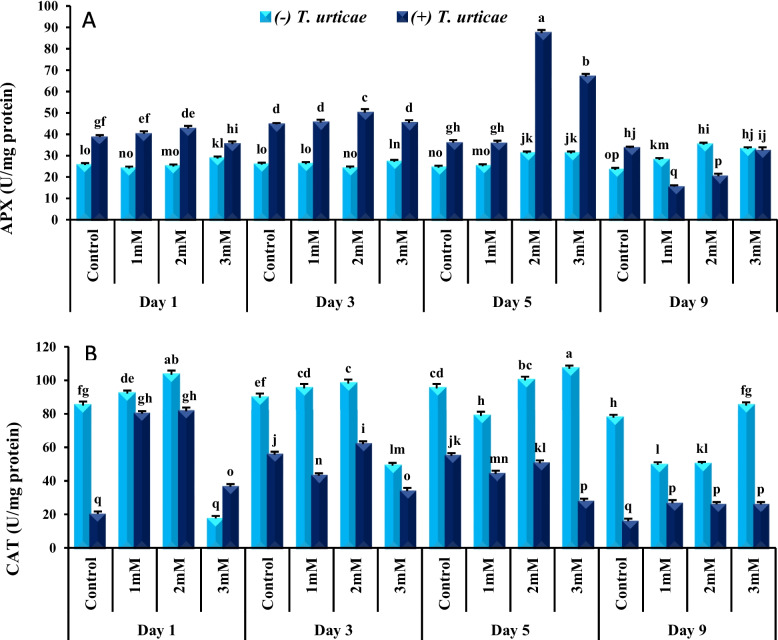
Ascorbate peroxidase (APX) (**A**) and catalase (CAT) (**B**) activity in Spadana cucumber leaves in response to *Tetranychus urticae* and different spermine concentrations (1, 2, and 3 mM) treatments. Mean (± SD) was calculated from three replicates for each treatment. Bars with different letters are significantly different at *P* ≤ 0.05 applying LSD test

APX activity was higher in + *T. urticae* plants treated with 1, 2 and 3 mM spermine on day 1, 3 and 5 opposed to day 9 when APX activity in + *T. urticae* plants treated with spermine were lower or similar to—*T. urticae* plants (Fig. 2A). APX activity in + *T. urticae* plants treated with 1 mM spermine were similar to control + *T. urticae*. APX activity was at highest levels in + *T. urticae* plants treated with 2 mM spermine on day 1, 3 and 5 compare to control + *T. urticae*. On day 1 the lowest APX level was observed in + *T. urticae* plants treated with 3 mM spermine whereas APX activity increased significantly on those treated with 3 Mm spermine on day 5. On day 9 there was a significant reduction in APX activity in + *T. urticae* plants treated with 1 and 2 mM spermine however there was no significant difference between + *T. urticae* plants treated and 3 mM spermine with controls.

### The effect of spermine and *T. urticae* on cucumber CAT activity

As demonstrated in Fig. 2B *T* *. urticae* suppressed CAT activity in cucumber plants during the experiment. On day 1 in all + *T. urticae* plants treated with spermine CAT activity was increased compare to control + *T. urticae*. On day 1 spermine at 1 and 2 mM concentrations increased CAT activity in -*T. urticae* plants opposed to 3 Mm spermine which lowered CAT activity in -*T. urticae* plants. On day 3 and 5 spermine at 1 and 3 mM concentrations suppressed CAT activity compare to control in + *T. urticae* plants. However, + *T. urticae* plants treated with 2 mM spermine showed higher CAT activity on day 3 and unchanged on day 5 compared to control + *T. urticae*. On day 9, CAT activity was increased significantly in all + *T. urticae* plants treated with spermine compare to control + *T. urticae*.

### The effect of spermine on *T. urticae* developmental time, adult longevity and total life span in cucumber plant

The effect of spermine at different concentrations on the duration of life stages and total life span of *T. urticae* in cucumber plants were shown in Tables 1 and 2. Embryonic, protochrysalis and deutonymphal periods of this mite were not affected by different concentrations of spermine. Whereas, mites reared on cucumber plants treated with different concentrations of spermine showed a significant difference in larval, protonymphal, deutochrysalis and theliochrysalis periods. The longest larval period was related to 3 mM and the shortest was related to 1 and 2 mM spermine. The longest protonymphal period was recorded in 2 and 3 mM spermine whilst the shortest was seen in 1 mM concentration. The longest duration of deutochrysalis was observed in 2 mM treatment whereas, the shortest belonged to 1 mM. The longest theliochrysalis period of *T. urticae* was related to 2 mM concentration which was significantly different from control. There was no difference in preadult time among different treatments, however, the longest adult longevity and total life span was observed in 1 and 3 mM spermine concentrations.

**Table 1:** The mean (± SE) duration of different developmental stages and adult longevity of *Tetranychus urticae* on cucumber plants treated with different concentrations of spermine

**Treatment**	**Egg** **(d)**	**Larva** **(d)**	**Protochrysalis (d)**	**Protonymph (d)**	**Deutochrysalis (d)**	**Deutonymph (d)**	**Theliochrysalis (d)**	**Preadult** **(d)**
Control	3a	0.97 ± 0.08ab	0.81 ± 0.04a	0.82 ± 0.04ab	0.63 ± 0.03ab	1.06 ± 0.05a	0.86 ± 0.03b	9.05 ± 0.12a
Spm 1 mM	3a	0.80 ± 0.07b	0.85 ± 0.04a	0.77 ± 0.04b	0.58 ± 0.03b	1.15 ± 0.07a	0.94 ± .0.03ab	8.90 ± 0.11a
Spm 2 mM	3a	0.82 ± 0.07b	0.75 ± 0.05a	0.93 ± 0.06a	0.71 ± 0.04a	1.08 ± 0.05a	0.98 ± 0.02a	9.05 ± 0.11a
Spm 3 mM	3a	1.12 ± 0.13a	0.75 ± 0.05a	0.94 ± 0.05a	0.64 ± 0.03ab	1.08 ± 0.05a	0.94 ± 0.03ab	9.16 ± 0.16a

The means followed by different letters in each column are significantly different (paired-bootstrap at 5% significance level)

**Table 2:** The mean (± SE) adult pre-oviposition, total pre-oviposition and oviposition, periods, female fecundity, total and adult longevity of *Tetranychus urticae* on cucumber plants treated with different concentrations of spermine

Treatment	APOP (d)	TPOP (d)	Ovi. period (d)	Fecundity	Total longevity (d)	Adult (d)
Control	0.62 ± 0.04a	9.68 ± 0.15a	8.96 ± 0.88b	70.23 ± 7.07b	22.71 ± 0.90b	13.65 ± 0.88b
Spm 1 mM	0.67 ± 0.04a	9.64 ± 0.07a	14.43 ± 1.33a	121.81 ± 13.26a	26.00 ± 1.16a	17.10 ± 1.18a
Spm 2 mM	0.68 ± 0.05a	9.79 ± 0.27a	11.27 ± 1.00ab	93.88 ± 9.89ab	23.20 ± 0.93ab	14.14 ± 0.97ab
Spm 3 mM	0.76 ± 0.07a	10.09 ± 0.47a	11.60 ± 1.26ab	92.36 ± 10.25ab	25.97 ± 1.28a	16.81 ± 1.33a

The means followed by different letters in each column are significantly different (paired-bootstrap at 5% significance level)

### The effect of spermine on *T. urticae* oviposition period and mean total fecundity in cucumber plant

The effect of spermine on APOP, TPOP, oviposition period and fecundity of *T. urticae* is represented in Table 2. The data from this experiment did not show a significant difference between the treatments in APOP and TPOP. The longest oviposition period belonged to 1 mM spermine and the shortest was observed in control. 1 mM spermine increased fecundity compared to control, but this was not the case in the higher concentrations of spermine (2 and 3 mM).

### The effect of spermine on *T. urticae* age specific survival rate in cucumber plant

**Fig. 3:**
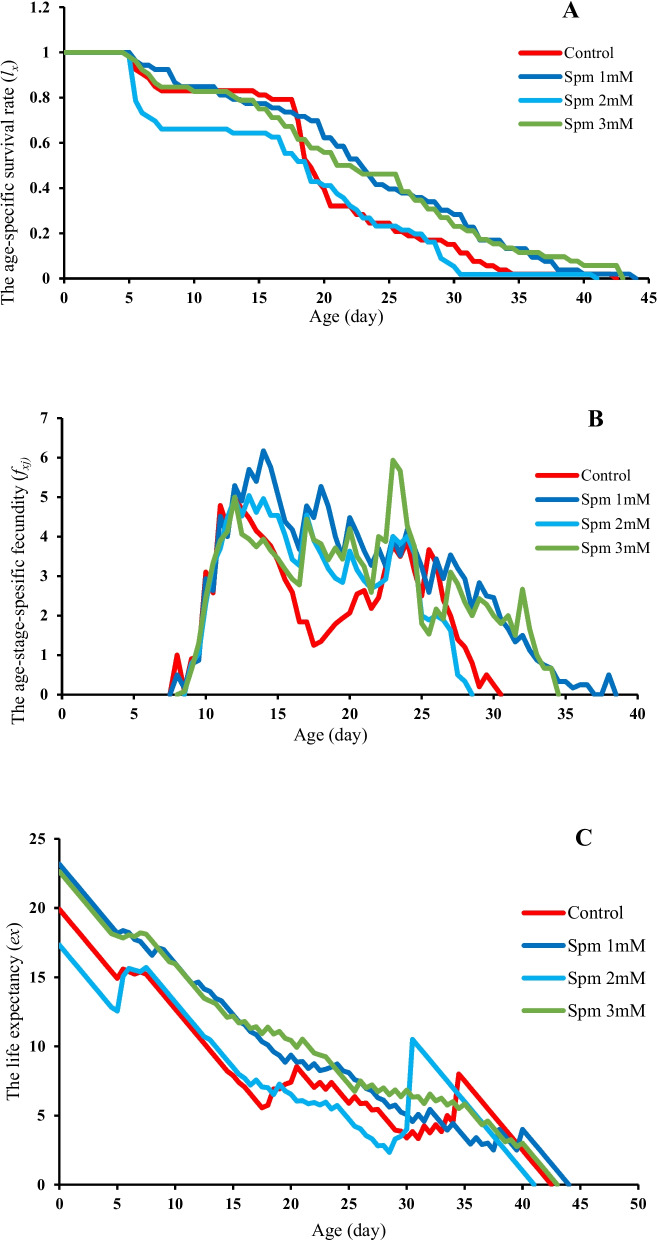
Age-specific survival rate (*l*_*x*_) (**A**), age-stage-specific fecundity (*f*_*xj*_) (**B**) and Life expectancy (*e*_*x*_) (**C**) of *Tetranychus urticae* on cucumber plants treated with different concentrations of spermine

The results of age specific survival rate (*l*_*x*_) are demonstrated in Fig. 3A. The graph from all treatments and control were matched from day 0 to 5. *l*_*x*_ in mites fed on 2 mM treated plants showed lowest readings throughout the experiment. *l*_*x*_ in 2 mM spermine treatments was reduced from day 5 to 8 (larval period) from 1 to 0.6. *l*_*x*_ in those treated with 2 mM spermine matched control from day 22 to 28.5 (adult period) at 0.6 before plummeting further reaching negligible level on day 30.5. The last death in plants treated with 2 mM spermine happened on day 41. Age specific survival rate in plants treated with 1 and 3 mM spermine was higher compare to 2 mM spermine and decreased slowly from day 5. The last death in plants treated with 1 and 3 mM spermine happened on day 44 and 43.

### The effect of spermine on *T. urticae* age-stage-specific fecundity in cucumber plant

The Age-stage-specific fecundity (*f*_*xj*_) is demonstrated in Fig. 3B. The *f*_*xj*_ started at day 7.5 to 8.5 in all plants including control and those treated with spermine and peaked and plummeted a number of time. Peaks for control and 1, 2 and 3 mM spermine concentrations were 4.97, 6.17, 5.03 and 5.66 eggs female^−1^ day^−1^ and the maximum was recorded in 1 mM spermine on day 14. The longest range of *f*_*xj*_ belonged to 1 mM spermine for 31 days and the shortest range of *f*_*xj*_ belonged to 2 mM spermine for 20 days. *f*_*xj*_ range for 3 mM spermine was 26.5 days which was longer than control but shorter than 1 mM spermine.

### The effect of spermine on *T. urticae* life expectancy in cucumber plant

The age specific life expectancy (*e*_*x*_) of *T. urticae* is represented in Fig. 3C. The *e*_*x*_ for control and 1, 2 and 3 mM spermine treatments were 19.91, 23.17, 17.33 and 22.64 days at the beginning of experiment. In general the lowest *e*_*x*_ from day 0 to 29.5 belonged to 2 mM spermine with downward slope but *e*_*x*_ for control and 2 mM spermine were compatible with each other from day 5.5 to 16.5. The *e*_*x*_ was increased from day 29.5 to 31 before falling down again and reaching 0 on day 41. The highest *e*_*x*_ until day 30 of experiment was seen in 1 and 3 mM spermine treatments and their curves were convoluted and almost compatible with a low downward slope. The *e*_*x*_ for control and 1, 2 and 3 mM spermine treatments reached 0 on day 42.5, 44, 41 and 43 respectively. The *e*_*x*_ for 2 mM spermine reached 0 in a shorter time. The *e*_*x*_ for 1 mM spermine was higher at the beginning of experiment compare to 2 mM treatment and control.

### The effect of spermine on *T. urticae* population parameters in cucumber plant

The population parameters of *T. urticae* reared on four experimental treatments were significantly different and demonstrated on Table 3. The intrinsic and finite rate of increase (*r*_*m*_ and *λ*) were affected by different concentrations of spermine and both followed the same trend in different treatments. The lowest value of *r*_*m*_ and *λ* was seen in 2 mM spermine whereas, 1 and 3 mM treatments were not significantly different from controls. The highest gross and net reproductive rates (*GRR* and *R*_0_) belonged to 1 mM spermine whereas, the lowest gross reproductive rate was seen in 2 mM spermine. Spermine affected mean generation time (*T*) in mites. This parameter was significantly higher in 1, 2 and 3 mM spermine treatments compare to controls.

**Table 3:** Population parameters (mean ± SE) of *Tetranychus urticae* on cucumber plants treated with different concentrations of spermine

Treatment	*GRR* (egg/individual)	*R*_0_ (egg/individual)	*r*_*m*_ (d^−1^)	*λ* (d^−1^)	*T* (d)
Control	75.95 ± 11.97b	45.05 ± 6.47b	0.2735 ± 0.0097a	1.3146 ± 0.0128a	13.91 ± 0.30b
Spm 1 mM	127.72 ± 15.42a	75.84 ± 11.52a	0.2826 ± 0.0099a	1.3266 ± 0.0131a	15.31 ± 0.21a
Spm 2 mM	91.17 ± 11.79ab	45.26 ± 7.85b	0.2586 ± 0.0102b	1.2951 ± 0.0155b	14.74 ± 0.22a
Spm 3 mM	98.21 ± 14.46ab	58.61 ± 8.95ab	0.2699 ± 0.0102ab	1.3099 ± 0.0133ab	15.08 ± 0.30a

The means followed by different letters in each column are significantly different (paired-bootstrap at 5% significance level)

## Discussion

### The effect of spermine and *T. urticae* on cucumber defensive biochemical parameters

**Fig. 4:**
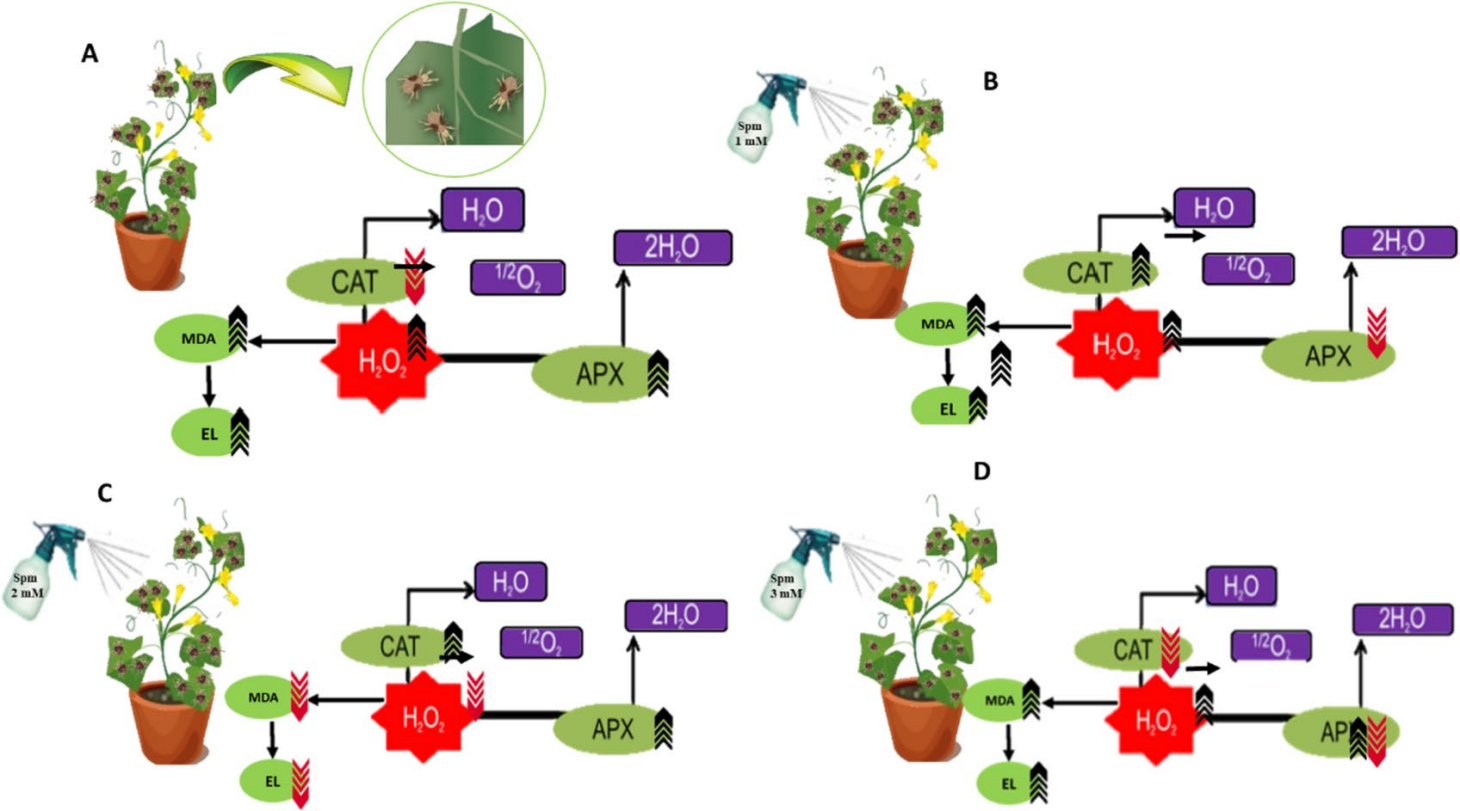
Antioxidant defense response in *Cucucmis sativus* leaves treated with *Tetranychus urticae* (**A**), *T. urticae* and 1 mM spermine (**B**), *T. urticae* and 2 mM spermine (**C**), *T. urticae* and 3 mM spermine (**D**). The induced defense is facilitated via defensive enzymes such as Catalase (CAT) and Ascorbate peroxidase (APX). CAT breaks down H_2_O_2_ to oxygen and water, and (APX) reduces H_2_O_2_ to water. MDA: malondealdehyde; EL: electrolyte leakage;  Indicates increase,  means decrease and   suggests fluctuation of parameters

As a group of living organisms, plants have developed various defense strategies to face biotic and abiotic stresses. The first response of plants in this situation is to activate the defense system, including production of ROS, especially H_2_O_2_. As mentioned before H_2_O_2_ does not only activate the defense response as a signaling molecule but also disrupts normal metabolic activity as a toxic compound [24, 36]. Therefore, feeding of herbivores like mites as biological stressors can increase the content of ROS in plants [27]. Our experiment demonstrated that feeding of *T. urticae* changed the physiological state of cucumber plant (Fig. 4) and led to an increase in H_2_O_2_ production in this plant.

Treating plants with polyamines, especially spermine and spermidine, inhibits ROS in abiotic stress conditions markedly [20]. There is not much information in literature regarding the effect of these compounds on plant–herbivore interaction. This experiment demonstrated spermine application at 2 mM concentration in mite infested plants leads to reduction in H_2_O_2_ content on day 1 and 2 similar to what is seen in abiotic stress conditions. On the other hand, spermine at 3 mM concentration increased H_2_O_2_ content in infested plants during the whole experiment as well as non-infested plants in day 5 and 9 only. It is noteworthy that 2 mM spermine increased H_2_O_2_ content of infested plants on day 5 and 9. Spermine catabolism in plants by Diamine oxidase (DAO) and Polyamine oxidase (PAO) leads to H_2_O_2_ production through two types of reactions; Terminal oxidation and Back conversion [3, 53]. Polyamine oxidation causes stress resistance by inducing the expression of H_2_O_2_ related certain signaling components and transcription factors. Polyamines are very toxic in high concentrations therefore, plant cells need to maintain the homeostasis of polyamines through biosynthesis, transfer or binding to other compounds [45]. Similar to H_2_O_2_, polyamines also function as a double-edged sword. Excessive accumulation of polyamines in plants can be harmful to cells, leading to toxicity and early cell death [28]. In the present study, the use of 3 mM spermine probably had such an effect. The possible explanation for earlier occurrence of H_2_O_2_ increment in *T. urticae* treatments could be the accumulation of H_2_O_2_ produced by mite activity, spermine catabolism, or intracellular interactions [26].

MDA represents an estimation for lipid peroxidation and fragmentation of polyunsaturated fatty acids of membrane phospholipids which is used as a common indicator to determine oxidative stress level [58]. MDA level in control + *T. urticae* plants, increased initially before falling down despite continued mite presence. It is felt that MDA reduction is due to antioxidant enzyme activity induction. This is because of significant inhibition of MDA content which reflects high antioxidant capacity, associating with high resistance or tolerance against herbivore attack [65]. MDA level was significantly higher in control plants with *T. urticae* compared to the control plants without mite on day1, 3, 5 and 9*.* Mite feeding begins with insertion of the stylet into the plant which disrupts cell membrane and therefore compounds such as MDA are released from destructed membrane [27].

In this experiment, the MDA trend was somewhat consistent with H_2_O_2_. ROS production leads to lipid peroxidation and produces unsaturated fatty acids including small hydrocarbon fragments such as ketone, MDA and related compounds [25, 27]. In infested plants treated with 1 mM spermine MDA level decreased in day 1, 3, and 5 whereas in plants treated with 2 mM spermine it only decreased on first and third day. There are various reports of the reduction of MDA content in plants under the influence of abiotic stresses and in presence of 1 mM and lower concentrations of polyamines [28, 32, 58]. There are limited information available in relation to biotic stresses. Therefore, it seems polyamines such as spermine at lower concentrations reduce MDA content against biotic and abiotic stresses. Spermine catabolism at 1 mM and to some extent at 2 mM concentrations produces appropriate level of H_2_O_2_ which possibly can activate antioxidant systems and strengthens plant cell wall under stress conditions [28]. By reducing the peroxidation of unsaturated fatty acids and finally maintaining the integrity of the membrane, MDA content decreases [58]. Similar to H_2_O_2_, 3 mM concentration of spermine in presence of *T. urticae* produced the highest MDA content. Yin et al*.* [62] demonstrated using 3 mM spermidine in *Cerasus humilis* (Bge.) Sok. exposed to abiotic stress (drought) has shown signs of toxicity in plant. MDA content increased significantly in all spermine treatments + *T. urticae* on day 9. Failure of antioxidant system in inhibiting ROS can increase MDA [5]. 1 and 2 mM spermine reduced MDA level and probably *T. urticae*- derived damage by fifth and third day respectively. It is perceived that increase in antioxidant activity induced by spermine rises plant resistance to stressor i.e., *T. urticae.* Antioxidant activation and increase in plant resistance to stressors comes with potential costs for the host plant. It is perceived these potential costs may expand plant’s ability to control MDA. One possible solution would be to empower the plant with repeating spermine treatment on day 9.

Electrolyte leakage (EL) is an indicator of plants cellular response to stressors and quantifies stress-induced damage and plant's tolerance. Under stress condition, ROS open the K^+^ valves which cause leaking K^+^ out of cells [17]. The current experiment demonstrated *T. urticae* increases EL similar to H_2_O_2_ and MDA contents. Gangopadhyay et al. [24] also showed cellular membrane damage and subsequent EL increases in *Plumbago zeylanica* L. plant infested with *T. macfarlanei* Baker & Pritchard. EL changes during our experiment were almost similar to H_2_O_2_ and MDA changes. Treatment of cucumber plants with 1 and 2 mM spermine reduced EL caused by mite feeding on day 1 and 3. Spermine acts as a polycation at cellular pH. This polyamine can help maintaining the stability and permeability of cell membranes through electrostatic binding to proteins and negatively charged phospholipid head group [62]. Other studies showed applying 100 μM putrescine to wheat under drought stress and 10 mM putrescine to tomato under cold stress reduces EL [29, 33]. The current experiment showed 3 mM spermine was unable to lower EL nor H_2_O_2_ and MDA.

In our experiment *T. urticae* increased APX activity in cucumber plants. Santamaria et al. [52] showed *T. urticae* increased APX activity in *Arabidopsis thaliana*(L.) Heynh.. APX activity in *Glycine max* (L.) Merr. leaves increased significantly after infection with *Aphis craccivora* Koch [41]. Considering the higher level of H_2_O_2_ in mite-infested treatments compared to mite-free ones, it seems logical to increase the activity of antioxidant enzymes such as APX. Peroxidase enzymes, including APX, catalyze reduction of peroxides such as H_2_O_2_ to water in presence of electron receptors and play an important role in lowering oxidative damage [61]. Peroxidase enzymes such as cytosolic APX control H_2_O_2_ accumulation in plants. This enzyme plays an important role in defense mechanism against a wide range of stressors, including environmental factors such as drought, salinity, intense light, high and low temperatures, and pathogens [41]. The close relationship between early induction of APX and reduction in H_2_O_2_ content confirms the defensive role of this enzyme in cucumber plants against *T. urticae*.

Plant bearing *T. urticae* treated with 2 mM spermine showed highest level of APX activity on day1, 3 and 5. Peroxidase enzymes participate in many physiological processes including embryogenesis, auxin catabolism, lignification, cell wall destruction and defense against biotic and abiotic stressors [41]. As well as above effects, increase in peroxidase activity causes reduction in herbivore feeding as a result of phenolic compound oxidation. Derivatives of these compounds form active quinones in damaged tissues disrupt the absorption of nutrients for sucking pests through polymerization reaction [22, 40]. In addition, quinone compounds are directly toxic for herbivores [18, 23]. Peroxidase enzymes modify cell wall structure causing plant resistance to pests [56]. These enzymes strengthen defense barrier of plant cell wall by initiating lignification process in order to prevent pests' stylet penetration into plant cells [13]. Here despite our expectation, the strong increase of H_2_O_2_ on the day 9 in the presence of *T. urticae* and spermine did not result in higher APX activity. APX can reduce H_2_O_2_ content but on the other hand, it can be deactivated by H_2_O_2_. This enzyme plays a role in different cellular processes of the plant and its instability to H_2_O_2_ can be important in relation to such functions. APX deactivation causes limitations in resistance to stressors [31].

Antioxidant enzyme activity usually increases under biotic and abiotic stresses however, in our study CAT activity decreased in presence of *T. urticae*. Similar to our observation CAT activity was decreased in bean plants bearing *T. urticae* and also in *Vigna mungo* (L.) Hepper and *Micrantha mikania* Kunth plants infested with *B. tabaci* [21, 55, 64]. This conflict could be explained by variable metabolic pathways removing ROS or binding of H_2_O_2_ molecules to the active site of CAT leading to its deactivation [83]. The other explanation could be the suppression of plant's defense system by *T. urticae* [51].

Treatment of *T. urticae* infested cucumber plants with 2 mM spermine increased CAT activity during the entire duration of experiment. Spermine increases plant tolerance to stress via increasing antioxidant enzymes such as CAT [53]. It was noted that the 3 mM spermine induced the lowest CAT activity on day1, 3 and 5 of experiment when compared to other concentrations. Catalase activity decreased significantly on day 9 compared to previous days in *T. urticae* infested plants treated with all three spermine concentrations. This could be explained by catalase inhibition due to H_2_O_2_ surge on day 9 [28].

### The effect of spermine on *T. urticae* life table parameters in cucumber plants

**Fig. 5:**
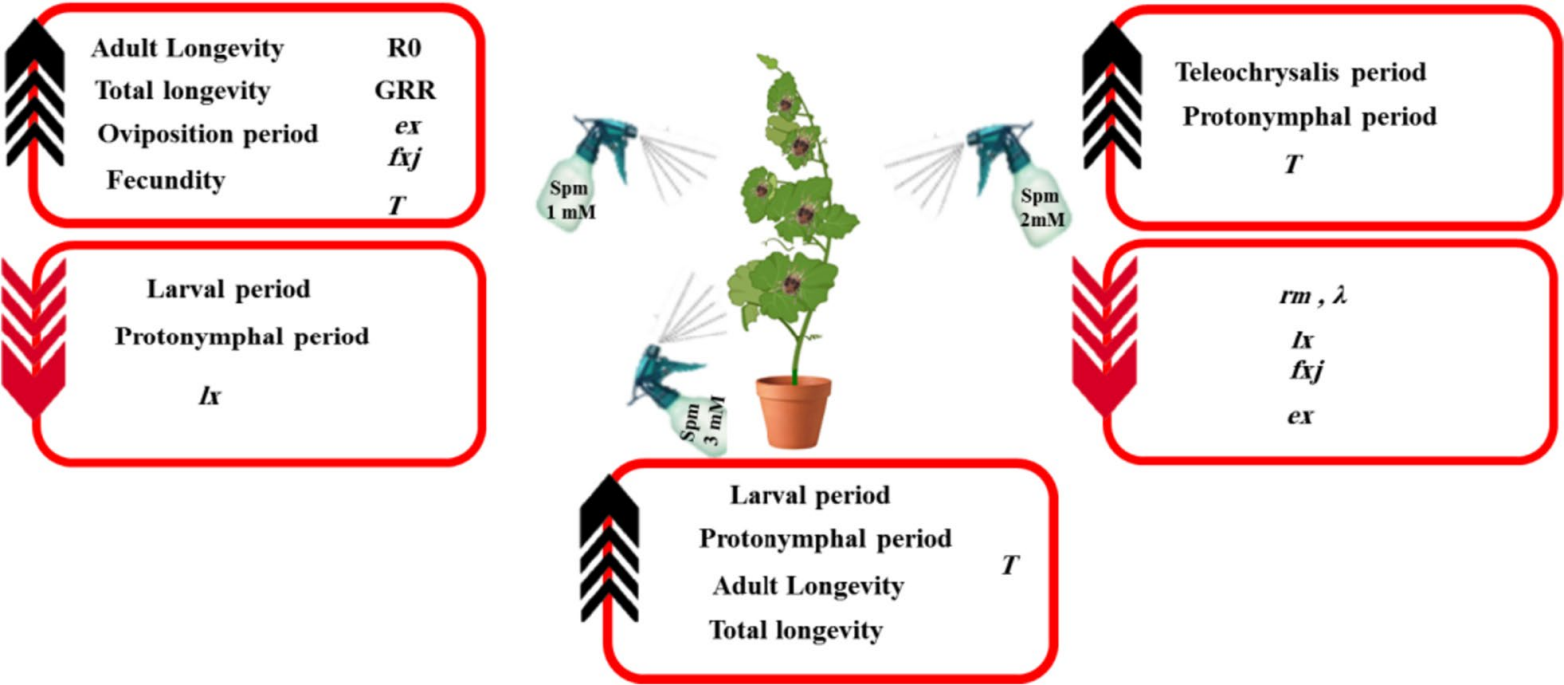
Bottom-up effect of spermine different concentrations on *Tetranychus urticae* life table parameters in cucumber plant. The black and red arrows represent increase and decrease effect respectively. *e*_*x*_: age specific life expectancy; *f*_*xj*_: age-stage-specific fecundity; *l*_*x*_: age specific survival rate; *r*_*m*_ and *λ*: the intrinsic and finite rate of increase respectively; *GRR* and *R*_0_): gross and net reproductive rates respectively; *T*: mean generation time

The present research demonstrated that spermine application on cucumber plants can affect demographic parameters of *T. urticae* feeding on them (Fig. 5). Lengthening of mite's growth periods can indicate poor nutritional quality or the presence of secondary metabolites in the host plant [48]. Comparison of *T. urticae* larval and protonymph periods showed the use of spermine at lower concentration (1 mM) provides a more suitable host for this mite opposed to higher concentrations (2 and 3 mM) which change plant physiology to mite's detriment. Mites feeding on plants treated with 2 and 3 mM spermine had the longest protonymph periods whereas 1 mM spermine induced the shortest. The larval stage of *T. urticae* was noted to be prolonged by 3 mM spermine in our experiment. Polyamines are classified in group of plant hormones because of their role in increasing the tolerance of plants against stress and regulating their growth and development. Senthil-Nathan et al*.* [54] showed foliar spraying of 2.5 and 5 mM jasmonic acid on rice leaves prolongs instar period of *Nilaparvata lugens* (Stal).

Our experiments showed 1 mM spermine had favorable effect on *T. urticae* by not only shortening of preadult period but also prolonging adult longevity. Exogenous polyamines increase endogenous polyamine level in plants providing higher food content for herbivores. Polyamines play an important role in developing of mucous membrane glands and gastric fluid promoting pest growth [35, 46]. 1 and 3 mM spermine prolonged adult longevity compared to control but 2 mM spermine didn't. It seems that increasing spermine concentration to 2 mM increases plant resistance to *T. urticae* especially during early growth stages but, this process changed when the concentration increased to 3 mM. It is assumed that 3 mM concentration is toxic for cucumber plant by weakening of defense mechanisms leading to elongation of mite adult longevity. Different concentrations of spermine can have dissimilar effects on the response of one sex to other sex pheromone and their mating [66]. On the other hand, mating can influence adult longevity [49]. As a result, mating and lifespan can differ in presence of various spermine concentrations. In the present study, the longest total longevity of mites belonged to 1 and 3 mM spermine. Other studies have also shown 1 mM spermine and spermidine prevents oxidative stress through induction of autophagy and increases *Drosophila* sp. total lifespan [19, 43]. 3 mM spermine does not provide a suitable host for *T. urticae* despite it prolonged mite’s total longevity and larval period.

The current experiment showed that 1 mM spermine was the only concentration which makes a meaningful positive impact on mite's fecundity. Whilst the direct role of polyamines on fertilization is not known, their presence is essential in reproduction process [6]. Polyamines are important for natural vitellogenesis and absence of endogenous polyamines ceases RNA production for embryogenesis [35]. Mysarla et al*.* [46] showed feeding silkworm larvae from leaves treated with 1 mM spermine and 0.05 mM spermidine increases egg production by 51% and 43% respectively. These polyamines increase fertility, testes growth, and early maturation.

Intrinsic rate of population increase (*r*_*m*_) is a useful parameter to interpret growth potential in insect populations exposed to different treatments or nutrients and is regarded as one of the most important biological and demographic indicators in pests [12]. Our experiment demonstrated 1 mM spermine created high fecundity and total longevity in *T. urticae* which led to a high level of *r*_*m*_. Amongst spermine treatments the highest values for *r*_*m*_, *λ*, *GRR* and *R*_0_ were observed in 1 mM spermine concentration. Considering above, it seems that 1 mM spermine has provided a more suitable host compare to other concentrations. On the other hand, 2 mM spermine induced resistance of host against *T. urticae* considering lower survival rate (*l*_*x*_), reproductive range (*f*_*xj*_), life expectancy (*e*_*x*_) and early death of the last individuals of *T. urticae*. 2 mM spermine was the only treatment that lowered mite *r*_*m*_ and *λ* compare to control.

## Conclusion

Our results showed that spermine significantly alters *Cucumis sativus—Tetranychus urticae* interaction. It seems that the effect of spermine as a pseudo-hormonal compound is not completely consistent with hormonal compounds such as jasmonic acid and affects the plant's physiology in a more complex way. Our findings demonstrated the effect of spermine on plant-mite interaction is different depending on spermine concentrations. 2 mM spermine was the only concentration that reduced cucumber sensitivity to *T. urticae* and 3 mM can be a toxic concentration for cucumber plants. Now a days, with high rate of acaricide resistance in *T. urticae*, severe damage caused by this mite, and no information on effect of polyamine application on herbivore arthropods, our findings can suggest a potential alternative for pest management that is of important agricultural and ecological implications.

## Methods

### Cucumber plants

Spadana commercial cultivar was used for mite colony establishment and experiments. Cucumber seeds were surface sterilized with 0.5% (v/v) Nalco, rinsed thoroughly with distilled water and kept in wet sterile napkin to germinate. After germination, the seeds were sown in a quartz sand-filled plastic tray. After two weeks, uniform-sized seedlings were transplanted into 8-L plastic containers with Hoagland’s solution. Plants were grown in a growth chamber under controlled conditions (photoperiod 16 h light/8 h dark; temperature 25 ± 5 °C; humidity 60%).

### Mite maintenance and leaf discs

In this experiment mite specimens were sourced from *T. urticae* colony in Isfahan University of technology entomology lab. Mites were reared on Spadana cultivar cucumber plants in the laboratory (photoperiod 16 h light/8 h dark; temperature 21 ± 1 °C; humidity 60%) for use in the experiments. To accomplish the experiments, fully expanded young leaves were used for the leaf disc preparation. The leaves were cut into 4 cm pieces and placed on top of a water-soaked cotton webril in 90 mm diameter Petri dishes. During the experiments, these leaf discs were prepared for each treatment and the mites were transferred on them.

### Spermine treatment

Eight leaf plants were used to investigate the effect of spermine on biochemical parameters related to antioxidant defense against *T. urticae*. Each treatments with its three replicate were established as: –*T. urticae* (control = no spermine, 1, 2 and 3 mM spermine) and + *T. urticae* (control = no spermine, 1, 2 and 3 mM spermine). The plants were sprayed with water (control) or different concentrations of spermine treatments. Tween-20 (0.01%, v/v) was used both in spermine solution and water as a surfactant to increase adsorption, ensuring both sides of the leaves were all covered with solution. For biochemical experiments, eight-leaf cucumber plants were sprayed by either water or different concentrations of spermine. One hour after spraying ten 12-h-old mites (5 male and 5 female) were placed on each leaf of the plants in + *T. urticae* treatments. The leaves of cucumber plants were used for the experiments on the first, third, fifth and ninth days after spraying the solution and settling the mites.

To make the conditions similar to previous experiment, eight leaf plants were used to investigate the effect of spermine on *T. urticae* life table parameters too. The leaves of these plants were used to prepare leaf disks after being treated with water or different concentrations of spermine as described above. The leaf discs used for mite feeding were changed every two days.

### Hydrogen peroxide (H_2_O_2_) concentration

H_2_O_2_ content was determined using Loreto and Velik-ova (2001) method [39]. Frozen leaves were extracted on ice bath using 2 mL of 0.1% (w/v) trichloroacetic acid (TCA) and subsequently, the homogenate was centrifuged at 4 °C for 15 min at 12,000 g. 0.5 mL of 10 mM phosphate buffer (pH 7) and 1 mL of 1 M potassium iodide (KI) solution were added to 0.5 mL of the supernatant extract. The absorbance of the samples was recorded at 390 nm by spectrophotometer (Unico, Model UV-2100, USA). The concentration of the samples was determined using standard curve. The blank contained 1 mL of 10 mM potassium phosphate buffer and 1 mL of 1 M KI solution.

### Lipid peroxidation determination

MDA as the most abundant aldehyde resulting from lipid peroxidation was determined in cucumber leaves, as per Heath and Parker (1968) [30]. Frozen leaf material was ground in liquid nitrogen in a mortar-pestle. 0.1 g leaf tissue powder was added into centrifuge tube and mixed with 1 mL of 0.1% (w/v) TCA. The extract was centrifuged at 12,000 g for 20 min at 4 °C. 1 mL of supernatant was transferred to a new test tube and mixed with 3 mL TCA 20% (w/v) containing 0.5% thiobarbituric acid (TBA). The mixture was boiled at 95 °C for 30 min before immediately placed on ice to stop the reaction. The samples were subsequently centrifuged at 10,000 g for 5 min and the spectrophotometry was applied to define the MDA content. 0.5 mL of TCA and 1 mL of TCA-TBA were used as blank. The spectrophotometry was applied at 532 and 600 nm to define the MDA content (instrument model???).

### Electrolyte leakage determination

The electrolyte leakage of leaf samples was quantified based on Arora et al. (1992) [4]. Briefly, leaf discs (1 cm in diameter) were cut from the leaves and thoroughly rinsed with distilled water to release material from the wounded edges. Leaf discs were placed in 50 falcon tubes with caps containing 10 mL of deionized water. The contents were slowly washed for 24 h at room temperature using a rotary shaker (100 rpm). Then, an electrical conductivity meter (CC-501, Elmetron, Zabrze, Poland) was employed to measure the solution Electric Conductivity (EC1). The samples were boiled for 10 min at 100 °C and second reading was taken after cooling the solution to room temperature (EC2). The electrolyte leakage was calculated as EC1/EC2 and presented as a percentage.

### Antioxidant enzyme activity assays

Catalase (CAT) and ascorbate peroxidase (APX) extraction were conducted according to Liu et al. (2007) [38]. 100 mg of frozen leaf sample was homogenized in a blender with 1 mL ice-cold phosphate buffer (pH 7.0). The extract was centrifuged at 15,000 g for 20 min at 4 °C and the supernatant was immediately used to measure antioxidant enzyme activity and protein content. CAT activity was measured using the conversion rate of H_2_O_2_ to water and oxygen molecules at 240 nm. The decrease in absorbance was recorded by spectrophotometer (instrument model ?????) for 3 min at every 30 s. A mixture of 2 mL of phosphate buffer and 1 mL of 30 mM H_2_O_2_ was used as a blank. APX activity was calculated according to Cakmak and Marschner (1992) by monitoring the rate of ascorbate oxidation [11]. The reaction mixture contained 25 mM phosphate buffer (pH 7.0), 0.1 mM Ethylenediaminetetraacetic acid (EDTA), and 1.0 mM H_2_O_2_, and 0.25 mM ascorbic acid. 0.1 mL of crude enzyme extract was added to initiate the reaction. The decrease in absorbance at 290 nm at interval of 15 s up to 3 min was followed. Leaves’ total protein concentration was measured by the method described by Bradford (1976) [10].

### Life table experiments

All experiments were carried out in an incubator in controlled conditions at photoperiod 16 h light/8 h dark; temperature 25 ± 1 °C; humidity 60%. Cucumber leaves contain mite eggs (within 12 h) were transferred to the separate petri dishes. The number of eggs used for each cultivar treatment was 50. When the larvae emerged, were placed individually on the upper side of different treatment cucumber leaf discs. Experimental units were checked every 12 h to record the different life stages. Leaf discs were replaced with treated fresh ones every two days. Each individual larva was recorded until death or adult emerging. Development and survivorship were recorded for all immature stages. Following the emergence of adults, females and males of each treatment were coupled and the number of laid eggs were recorded until the death of the last adult.

### Data analysis

Plant biochemical parameters related data were subjected to analysis of variance (ANOVA) based on a split-plot factorial experiment using the SAS software (Version 9.0; SAS Institute). Means were compared using the least significant difference (LSD) test at *P* < 0.05. The computer program TWOSEX-MSChart (Chi, 2023) was used in *T. urticae* life table data analysis [15]. To ensure more precise estimates, all of the standard errors were estimated by the bootstrap technique with 100,000 bootstrap number and then used the paired bootstrap test to compare the differences amongst treatments [50].

### Supplementary Information

**Additional file 1: Table S1. **Analysis of variance (mean square) for hydrogen peroxide (H_2_O_2_), malondialdehyde (MDA) content, electrolyte leakage (EL), ascorbate peroxidase (APX) and catalase (CAT) activity, in Spadana cucumber leaves in response to *Tetranychus urticae* and different spermine concentrations (1, 2 and 3 mM) treatments.

## Data Availability

The datasets generated and analyzed during the current study are available from the corresponding author.

## Abbreviations

APOP: Adult preoviposition period
APX: Ascorbate peroxidase
CAT: Catalase
DHAR: Dehydroascorbate reductase
EC: Electric Conductivity
EDTA: Ethylenediaminetetraacetic acid
EL: Electrolyte leakage
*e*_*x*_: Life expectancy
*f*_*xj*_: Age-stage-specific fecundity
GR: Glutathione reductase
*GRR*: Gross reproductive rates
GST: Glutathione-S-transferase
GPX: Guaiacol peroxidase
H_2_O_2_: Hydrogen peroxide
KI: Potassium iodide
*l*_*x*_: Age specific survival rate
MDA: Malondialdehyde
MDHAR: Monodehydroascorbate reductase
*R*_0_: Net reproductive rates
*r*_*m*_: Intrinsic rate of population increase
ROS: Reactive oxygen species
SOD: Superoxide dismutase
*T*: Mean generation time
TBA: Thiobarbituric acid
TCA: Trichloroacetic acid
TPOP: Total preoviposition period
*λ*: Finite rate of population increase
