# Supplementary material for: The effect of spermine on Tetranychus urticae-Cucumis sativus interaction
Source: BMC Plant Biol. 2023 Nov 17;23:575. doi: 10.1186/s12870-023-04573-5 (PMC10655325; doi:10.1186/s12870-023-04573-5)
Supplement: Supplementary file 1 — Additional file 1: Table S1. Analysis of variance (mean square) for hydrogen peroxide (H2O2), malondialdehyde (MDA) content, electrolyte leakage (EL), ascorbate peroxidase (APX) and catalase (CAT) activity, in Spadana cucumber leaves in response to Tetranychus urticae and different spermine concentrations (1, 2 and 3 mM) treatments. [file 12870_2023_4573_MOESM1_ESM.docx]

**Table S1**. Analysis of variance (mean square) for hydrogen peroxide (H_2_O_2_), malondialdehyde (MDA) content, electrolyte leakage (EL), ascorbate peroxidase (APX) and catalase (CAT) activity, in Spadana cucumber leaves in response to *Tetranychus urticae* and different spermine concentrations (1, 2 and 3 mM) treatments.

| **Sources** | **df** | **H_2_O_2_** | **MDA** | **EL** | **APX** | **CAT** |
| --- | --- | --- | --- | --- | --- | --- |
| **Tet** | 1 | 278.30^**^ | 128.35^**^ | 1395.52^**^ | 5118.05^**^ | 32435.93^**^ |
| **Spm** | 3 | 31.22^**^ | 1.31^**^ | 62.51^**^ | 497.87^**^ | 2316.91^**^ |
| **Tet×Spm** | 3 | 4.49^*^ | 3.28^**^ | 60.32^**^ | 167.60^**^ | 492.70^**^ |
| **Rep(Tet×Spm)** | 16 | 2.52^*^ | 0.36^n.s^ | 3.14^n.s^ | 2.96^n.s^ | 11.50^n.s^ |
| **Time** | 3 | 43.16^**^ | 18.09^**^ | 110.02^**^ | 898.81^**^ | 3051.96^**^ |
| **Time× Tet** | 3 | 28.88^**^ | 13.90^**^ | 35.51^**^ | 1193.80^**^ | 1032.44^**^ |
| **Time×Spm** | 9 | 8.80^**^ | 2.22^**^ | 28.82^**^ | 287.89^**^ | 1881.76^**^ |
| **Time×Tet×Spm** | 9 | 7.92^**^ | 2.03^**^ | 15.53^**^ | 266.90^**^ | 974.35^**^ |
| **Error** | 48 | 1.12 | 0.21 | 3.26 | 3.68 | 9.91 |
| **CV%** |  | 13.13 | 16.45 | 5.34 | 5.53 | 5.14 |

df, degrees of freedom; Tet, *Tetranychus.urticae*; Spm, spermine; Rep, repetition; ns, non-significant, ^*^and ^**^ significant at *P*<0.05 and *P*<0.01, respectively.
